# RNA-Seq Count Data Modelling by Grey Relational Analysis and Nonparametric Gaussian Process

**DOI:** 10.1371/journal.pone.0164766

**Published:** 2016-10-26

**Authors:** Thanh Nguyen, Asim Bhatti, Samuel Yang, Saeid Nahavandi

**Affiliations:** 1 Institute for Intelligent Systems Research and Innovation, Deakin University, Victoria, Australia; 2 Department of Emergency Medicine, Stanford University, California, United States of America; Cleveland Clinic Lerner Research Institute, UNITED STATES

## Abstract

This paper introduces an approach to classification of RNA-seq read counts using grey relational analysis (GRA) and Bayesian Gaussian process (GP) models. Read counts are transformed to microarray-like data to facilitate normal-based statistical methods. GRA is designed to select differentially expressed genes by integrating outcomes of five individual feature selection methods including two-sample t-test, entropy test, Bhattacharyya distance, Wilcoxon test and receiver operating characteristic curve. GRA performs as an aggregate filter method through combining advantages of the individual methods to produce significant feature subsets that are then fed into a nonparametric GP model for classification. The proposed approach is verified by using two benchmark real datasets and the five-fold cross-validation method. Experimental results show the performance dominance of the GRA-based feature selection method as well as GP classifier against their competing methods. Moreover, the results demonstrate that GRA-GP considerably dominates the sparse Poisson linear discriminant analysis classifiers, which were introduced specifically for read counts, on different number of features. The proposed approach therefore can be implemented effectively in real practice for read count data analysis, which is useful in many applications including understanding disease pathogenesis, diagnosis and treatment monitoring at the molecular level.

## Introduction

Discovery of genes that are differentially expressed is helpful in gaining insights into disease pathogenesis, and discovering biomarkers for diagnosing and predicting the clinical status of patients. Identifying gene biomarkers is often performed using DNA microarray, which measures gene expression of the entire human genome. DNA microarray technology however suffers from the cross-hybridization procedure that yields noisy gene expression profiles. RNA sequencing (RNA-seq) has been emerging as a favorite method against the microarray technology [1]. RNA-seq is a technique that is capable of generating RNA-seq count data based on the next generation sequencing (NGS) technologies. The count data are structured as a table, which reports the number of sequence fragments assigned to each gene for each sample. RNA-seq is increasingly preferable to DNA microarray because it produces low background noise count data that allow detecting transcripts at low expression levels [2, 3]. With the decreasing cost of sequencing, the use of RNA-seq for differential expression analysis has been increased rapidly. NGS is able to measure the expression levels of tens of thousands of transcripts simultaneously. Such information is useful for developing expression-based classification algorithms to determine the diagnostic category of disease, for example cancers [4, 5].

**Fig 1:**
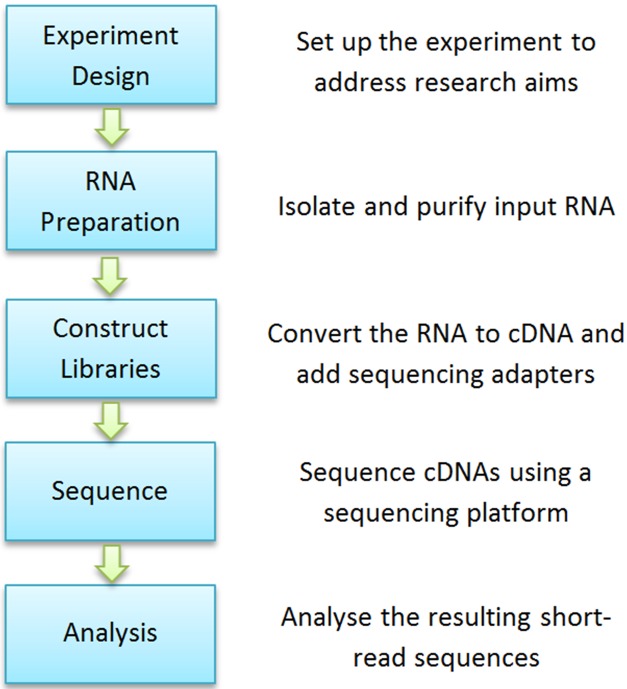
Basic steps of a typical RNA-seq experiment.

Fig 1 shows basic steps of a typical RNA-seq experiment. Specifically, an RNA-seq experiment normally requires a task of making a collection of cDNA fragments that are flanked by sequencing adapters. This library of cDNA fragments is then sequenced using a short-read sequencing platform. This step results in millions of short sequence reads that correspond to individual cDNA fragments.

As the RNA-seq technology provides count data, much interest has focused on statistical methods designed specifically for discrete counts, for example approaches using Poisson and negative binomial (NB) distributions. Witten et al. [6] introduced a Poisson linear discriminant analysis for modelling RNA-seq data. Alternatively, a specific nonlinear Poisson transformation was proposed in [7] and applied to the mRNA expression model to synthetically generate the RNA-seq data. Likewise, several over-dispersed Poisson models were introduced in [8–10]. A comparison of methods and software packages for detecting differential expression in RNA-seq studies was presented in [11, 12].

Due to the overdispersion issue, i.e. the variances are likely to exceed the means for a considerable number of genes [13], the Poisson distribution may not be suitable for modelling RNA-seq profiles when there are biological replicates. The NB distribution is therefore more general because it can mitigate this issue [14].

Robinson and Smyth [15] presented a quantile-adjusted conditional maximum likelihood estimator for the dispersion parameter of the NB distribution accompanying by the R package edgeR, which was detailed in [16]. Anders and Huber [17] proposed a method along with the DESeq package using the NB distribution with variance and mean linked by local regression. Hardcastle and Kelly [18] developed the algorithm baySeq that uses an empirical Bayes approach to discover patterns of differential expression by assuming a NB distribution for the data. Likewise, Wu et al. [19] introduced a shrinkage estimate of the dispersion parameters of the NB model for RNA-seq data. This estimator characterizes the variation in gene-specific dispersion and provides a better detection of differential expression genes compared with edgeR and DESeq. Love et al. [20] presented DESeq2, a successor to the DESeq method, to facilitate a more quantitative analysis of comparative RNA-seq count data using shrinkage estimators for dispersion and fold change.

Modelling sequencing data using count distributions is mathematically intractable and complicated because of the presence of extreme values, high skewness and the mean-variance dependency. Therefore, an alternative approach has emerged by using transformation procedures for the count RNA-seq data and applying normal-based microarray-like statistical methods. This reduces the disadvantages relating to the mathematical intractability of count distributions compared to the normal distribution and opens access to a wide range of known algorithms developed for microarray data. Several prevalent methods include logarithm transformation [3], variance-stabilizing transformation (VST) [17], TMM transformation [21], regularized logarithm [20], and variance modelling at the observation level “voom” method [22]. voom was verified and demonstrated that it performs as well or better than existing RNA-seq methods. This paper therefore promotes the use of voom method to process the RNA-seq data.

Using the voom transformation, we introduce an aggregate feature selection method based on the grey relational analysis (GRA) technique [23] to deal with transformed RNA-seq data. Compressed feature subsets obtained by the filter GRA method are fed into the Bayesian nonparametric Gaussian process (GP) models [24] for classification. Benchmark sequencing datasets are used to validate and show the significant dominance of the proposed approach against competing methods. We also perform rigorous statistical significance test to ensure the conclusions driven out of this study are valid and general. Next section presents in detail the proposed methodology and motivations of using GRA and GP methods.

## Methods

**Fig 2:**
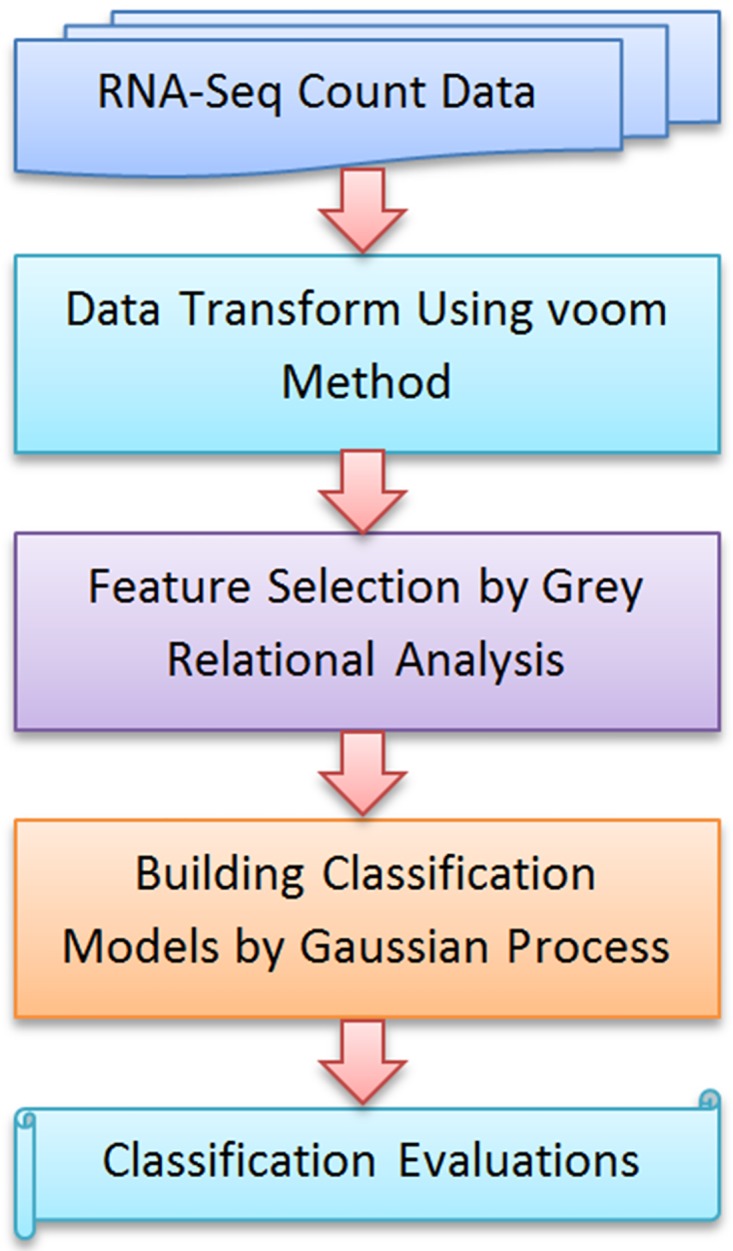
Proposed methodology for analyzing RNA-seq count data.

The proposed methodology for analysis of RNA-seq read counts is graphically presented in Fig 2. One of the basic tasks in the analysis of RNA-seq count data is the detection of differentially expressed genes [25]. In this paper, the RNA-seq read counts are first transformed using the voom method [22]. The transform alleviates the typical skewness, dependency between mean and variance or extreme values of RNA-seq data. After the transformation, RNA-seq data can be treated as if it was microarray data. This means that any normal-based methods or gene set testing procedures can be applied to RNA-seq data. We then design the GRA-based aggregate feature selection method that combines outcomes of five individual methods including two-sample t-test, entropy test (known as Kullback-Liebler distance or divergence) [26], Bhattacharyya distance [27], Wilcoxon test [28] and receiver operating characteristic (ROC) curve [29] to select significant genes as biomarkers. GRA-based method performs as a filter approach based on the assumption that the features are independent. This assumption is often made for high-dimensional low-sample data as there are too few observations available to be able to effectively estimate the dependence structure among the features [6, 30–32].

Once discriminant feature subsets have been selected, they serve as inputs into the GP models for classification. GP is fast and computationally tractable based on analytic formulae. A GP is completely characterized by its mean and covariance functions but it is not limited by a parametric form. Being a nonparametric method, the number and nature of GP parameters are flexible and not fixed in advance but are determined from data. Therefore, uncertainty and complexity of RNA-seq data can be addressed effectively by GP models. Under the GP viewpoint, the models are transparent and hence amenable to interpretation compared to black-box methods such as neural networks [24]. Generalization capability of GP based on Bayesian formalism can yield high classification performance for RNA-seq data modelling. Details of the voom transform approach, GRA-based feature selection method and GP models are sequentially presented in the following subsections.

### RNA-seq data transformation

Raw RNA-seq data are assembled in integer read counts. Specific characteristics of RNA-seq data that concern analysts are the presence of extreme values, high skewness, and the mean-variance dependency (i.e. heteroscedasticity). Logarithm transformation is a prevalent method to eliminate RNA-seq extreme values [3]. The variance-stabilizing transformation (VST) proposed in [17] is also often used to deal with skewed RNA-seq data. Alternatively, Love et al. [20] introduced regularized logarithm to transform RNA-seq data to render them homoscedastic. Law et al. [22] proposed voom method that converts the counts to log-counts per million with associated precision weights. After this, the normal-based methods can be applied to RNA-seq data as if it was microarray data. Details of the voom method are presented in the Supplementary Materials section.

### GRA-based feature selection

GRA was introduced by Deng [33] and has been applied to solve multicriteria decision making (MCDM) problems in various fields [23, 34, 35]. GRA is part of grey system theory, which is capable of solving problems with complicated interrelationships between multiple factors and variables. We propose the use GRA as a filter feature selection approach that combines outcomes of individual methods including two-sample t-test, entropy test, Bhattacharyya distance, Wilcoxon test and ROC curve. Assume the MCDM problem has *m* alternatives and *n* criteria (attributes) where the *i*th alternative can be expressed as *Y*_*i*_ = (*y*_*i*1_,*y*_*i*2_,…,*y*_*ij*_,…,*y*_*in*_) where *y*_*ij*_ is the performance value of the criterion *j* of the alternative *i*. To formulate gene selection as an MCDM problem, we treat genes (features) as alternatives and individual methods as criteria. Therefore, there are *m* features corresponding to *m* alternatives. In this paper, *n* is equal to 5 as there are 5 individual methods corresponding to 5 criteria. Outcomes of individual methods are scores of every feature. For each individual method, we represent its scores as the performance values of corresponding features. The following presents steps of the GRA algorithm.

(1) Grey relational generating: This step is to translate the performance values of all alternatives into a comparability sequence. It normalizes data sequence for the experimental results within 0 and 1. If the larger target value of the original sequence is the better, then the normalization is performed by:

$$x_{ij}=\frac{y_{ij}-\min_{i}y_{ij}}{\max_{i}y_{ij}-\min_{i}y_{ij}}$$ (1)

Alternatively, if the smaller target value is the better then the original sequence is normalized by:

$$x_{ij}=\frac{\max_{i}y_{ij}-y_{ij}}{\max_{i}y_{ij}-\min_{i}y_{ij}}$$ (2)

where *x*_*ij*_ is the generating value of the grey relational analysis, $\min_{i}y_{ij}$ is the minimum value of *y*_*ij*_ among all alternatives *i* = 1,2,…,*m* and $\max_{i}y_{ij}$ is the maximum value of *y*_*ij*_.

(2) Define the reference sequence: Once the grey relational generating procedure is complete, all performance values are scaled into [0, 1]. An alternative will be the best choice if all of its performance values are equal to or close to 1. Therefore, we define the reference sequence *X*_0_ as (*x*_01_,*x*_02_,…,*x*_0*j*_,…,*x*_0*n*_) = (1,1,…,1,…,1) and then find the alternative whose comparability sequence is the closest to the reference sequence *X*_0_.

(3) Calculate the grey relational coefficient: This coefficient is used to determine how close *x*_*ij*_ to *x*_0*j*_. The larger the coefficient is the closer between *x*_*ij*_ and *x*_0*j*_. This coefficient can be computed by:

$$\delta \left(x_{0j},x_{ij}\right)=\frac{\Delta_{\min}+\alpha \Delta_{\max}}{\Delta_{ij}+\alpha \Delta_{\max}}$$ (3)

where Δ_*ij*_ = |*x*_0*j*_ − *x*_*ij*_|, $\Delta_{\min}=\min_{i,j}\Delta_{ij}$, $\Delta_{\max}=\max_{i,j}\Delta_{ij}$ and *α* is the distinguishing coefficient, *α* ∈ [0, 1]. The distinguishing coefficient may expand or compress the range of the grey relational coefficient. In this paper, we set *α* = 0.5 for all experiments.

(4) Calculate the grey relational grade between *X*_*i*_ and *X*_0_ using:

$$\phi \left(X_{0},X_{i}\right)=\overset{n}{\underset{j=1}{\sum }}w_{j}\delta \left(x_{0j},x_{ij}\right)$$ (4)

where *w*_*j*_ is the weight of attribute *j* and $\sum_{j=1}^{n}w_{j}=1$. The above equation is applied to all *m* alternatives *i* = 1,2,…,*m*. The grey relational grade represents the degree of similarity between the comparability sequence and the reference sequence. Therefore, if a comparability sequence for an alternative achieves the greatest grey relational grade with the reference sequence, that alternative is the best choice.

For the purpose of feature selection for classification, we rank alternatives (features) based on their corresponding grey relational grades. Features have the top grey relational grades are selected to form a feature set.

The next subsections scrutinize background of individual feature selection filter methods whose outcomes are used for the proposed GRA approach. These methods are accomplished by ranking features via scoring metrics. They are statistic tests based on two sets of data samples in the binary classification problem. The sample means are denoted as *μ*_1_ and *μ*_2_, whereas *σ*_1_ and *σ*_2_ are the sample standard deviations, and *n*_1_ and *n*_2_ are the sample sizes [36].

#### Two-sample t-test

The two-sample t-test is a parametric hypothesis test that is applied to compare whether the average difference between two independent sets of data samples is really significant. The test statistic is calculated by:

$$t=\frac{\mu_{1}-\mu_{2}}{\sqrt{\frac{\sigma_{1}^{2}}{n_{1}}+\frac{\sigma_{2}^{2}}{n_{2}}}}$$ (5)

In the application of t-test for gene selection, the test is performed on each gene by separating the expression levels based on the class variable. The absolute value of *t* is used to evaluate the significance among genes. The higher the absolute value, the more important is the gene.

#### Entropy test

Relative entropy, also known as Kullback-Liebler distance or divergence is a test assuming classes are normally distributed. The entropy score for each gene is computed using the following expression:

$$e=\frac{1}{2}\left[\left(\frac{\sigma_{1}^{2}}{\sigma_{2}^{2}}+\frac{\sigma_{2}^{2}}{\sigma_{1}^{2}}-2\right)+\left(\frac{1}{\sigma_{1}^{2}}+\frac{1}{\sigma_{2}^{2}}\right){\left(\mu_{1}-\mu_{2}\right)}^{2}\right]$$ (6)

After the computation is complete for every gene, genes with the greatest entropy scores are selected to serve as inputs to the classification techniques.

#### Bhattacharyya distance

The Bhattacharyya distance can be calculated from the standard deviation and mean of each class as follows:

$$BD=\frac{1}{4}\ln\left[\frac{1}{4}\left(\frac{\sigma_{1}^{2}}{\sigma_{2}^{2}}+\frac{\sigma_{2}^{2}}{\sigma_{1}^{2}}+2\right)\right]+\frac{1}{4}\left[\frac{{\left(\mu_{1}-\mu_{2}\right)}^{2}}{\sigma_{1}^{2}+\sigma_{2}^{2}}\right]$$ (7)

#### Wilcoxon method

The Wilcoxon rank sum test [28] is a test for equality of population locations (medians). The null hypothesis is that two populations enclose identical distribution functions whereas the alternative hypothesis states that two distributions differ regarding the medians. The normality assumption regarding the differences between the two samples is not required. That is why this test is used instead of the two-sample t-test in many applications when the normality assumption is concerned. The steps of the Wilcoxon test are summarized below [29]:

1. Assemble all observations of the two populations and rank them in the ascending order.
2. The Wilcoxon statistic is calculated by the sum of all the ranks associated with the observations from the smaller group.
3. The hypothesis decision is made based on the p-value, which is found from the Wilcoxon rank sum distribution table.

In the applications of the Wilcoxon test for gene selection, the absolute values of the standardized Wilcoxon statistics are utilized to rank genes.

#### Receiver operating characteristic curve

Denote the distribution functions of *X* in the two populations as *F*_1_(*x*) and *F*_2_(*x*). The tail functions are specified respectively *T*_*i*_(*x*) = 1 − *F*_*i*_(*x*), *i* = 1, 2. The ROC is given as follows:

$$ROC\left(t\right)=T_{1}\left[T_{2}^{-1}\left(t\right)\right],t\in \left(0,1\right)$$ (8)

and the area under the curve (AUC) is computed by:

$$AUC=\int_{0}^{1}ROC\left(t\right)dt$$ (9)

The larger the AUC, the less is the overlap of the classes. Genes with the greatest AUC therefore are chosen to form a gene set.

### Gaussian process models

A nonparametric GP is a generalization of the Gaussian probability distribution based on a Bayesian methodology. GP is defined as a collection of random variables, any finite number of which have a joint Gaussian distribution. GP can be used for function approximation problems including both classification and regression. In the regression problems, likelihood function is often assumed to be Gaussian, which combines with a GP prior to yield a posterior GP over functions. This exact Bayesian inference manipulation is analytically tractable. In classification problems, as the targets are discrete class labels, the Gaussian likelihood is therefore inappropriate. Therefore, an approximate inference is needed for classification problems. Several methods have been proposed that include Laplace’s method, Expectation Propagation (EP), variational approximations and Markov chain Monte Carlo (MCMC) modelling, e.g. see [37, 38].

The GP method for binary classification in this study is implemented by customizing the Gaussian processes for machine learning toolbox developed by Rasmussen and Nickisch [39]. Details of GP and its parameter settings are presented in the Supplementary Materials section.

### Experimental RNA-seq datasets

Two benchmark real datasets are utilized in this study for comparisons. We name the first dataset as “Mont-Pick” as it is obtained from a combination of two studies Montgomery et al. [40] and Pickrell et al. [41]. This data set is available through the ReCount RNA-seq database developed by Frazee et al. [42]. The data can be used to analyze differential expression between two ethnicities: the Montgomery group sequenced Utah people with ancestry from northern and western Europe (the HapMap CEU population) and the Pickrell group sequenced Yoruba residents in Ibadan, Nigeria (the HapMap YRI population). These two groups of ethnicities are treated as two separate classes in this study. There are 60 samples from the CEU group and 69 samples from the YRI group. A total of 52,580 genes are processed by which genes with zero counts in all samples are removed. The number of nonzero count genes of the Mont-Pick dataset is 12,984.

The second data set “cervical cancer” is available from Gene Expression Omnibus [43] under accession number GSE20592, which was utilized in [6, 44]. The data include 29 tumor and 29 non-tumor cervical tissue samples measured on 714 microRNAs, which are small RNAs with 18–30 nucleotides in length. The classification task is to distinguish between tumor and non-tumor samples. Details of the experimental datasets are presented in Table 1.

**Table 1:** Summary of RNA-seq datasets.

Datasets	Features	Samples	Classes
Mont-Pick [42]	12,984 genes	129	CEU/YRI
Cervical cancer [44]	714 microRNAs	58	tumor/non-tumor

**Fig 3:**
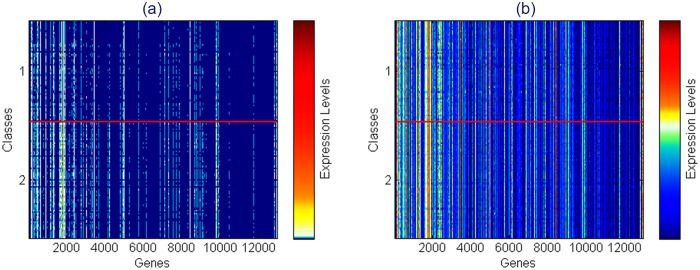
Heat maps showing expression levels of the Mont-Pick dataset (a) before and (b) after voom transform.

**Fig 4:**
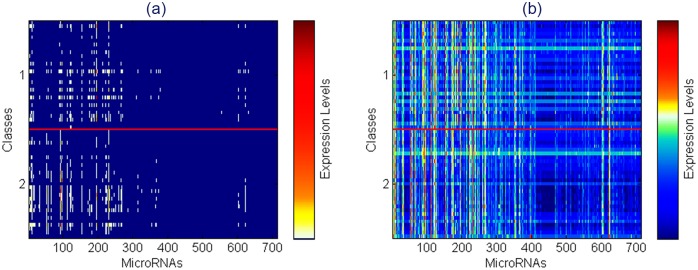
Heat maps of the expression levels in the cervical cancer dataset (a) before and (b) after voom transform.

In the Mont-Pick dataset, the CEU and YRI samples were sequenced by different groups using potentially different facilities. Therefore, the batch effect would be a factor that affects the performance comparisons of RNA-seq data analysis approaches. To deal with this issue, we have included the design option that addresses the batch effect in the voom transformation method, which is implemented in the limma package [45]. Figs 3 and 4 show pseudocolor heat maps of the expression levels before and after voom transformation for the Mont-Pick and cervical cancer datasets respectively. The x-axis represents genes or genomic features of interest whilst the y-axis represents data samples of different groups (classes). Both datasets used in this study have two classes of samples and the horizontal red line in every heat map divides the samples into the two classes. In each heat map, the corresponding color bar representing expression levels is plotted adjacent to the color map. The white color represents mid points, warm colors represent high expression levels and cool colors indicate low expression or sparse regions.

Before voom transform, the white region locates at the bottom of color bars in both datasets (see Figs 3a and 4a). This shows that read counts follow a positively skewed distribution, which would hinder the application of normal-based methods. Moreover, color maps of data before voom are almost blue with a very small proportion of warm color spots, which represent extreme values or outliers. The range of expression levels before voom transformation is extremely large, from 0 to 91,991 in the Mont-Pick dataset and from 0 to 476,438 in the cervical cancer dataset. The large blue areas in color maps represent sparse data, especially in Fig 4a. In contrast, the data after voom transformation is continuously distributed with the white region locates near the middle of color bars. In addition, the data after voom transform are less sparse than the original count reads as heat maps are more colorfully diversified with the combination of cool and warm colors. This demonstrates that the transformed data practically follow a normal distribution and can be processed by normal-based statistical methods.

### Performance evaluation metrics

To highlight the advantages of GRA-based feature selection method, we implement a number of competing methods for comparisons including ReliefF [46], iterative search margin based algorithm (Simba) [47], signal-to-noise ratio (SNR) [48], and information gain (IG) [49].

The following methods are also applied for comparisons with the designed GP classifier: k-nearest neighbors (kNN) [50], multilayer perceptron (MLP) [51], support vector machine (SVM) [52] and ensemble learning AdaBoost [53]. Specifically, the number of nearest neighbors in kNN is equal to 5 and SVM kernel function is the Gaussian radial basis function with the scaling factor of 1. MLP is constructed with two hidden layers and each layer comprises five nodes. AdaBoost uses a collection of individual learners that are 100 decision trees.

Four different performance metrics including accuracy rate, F1 score statistics (F-measure), AUC and mutual information (MI) are used to evaluate performance of classification methods. F-measure considers both the “Precision” (denoted as *Pr*) and “Recall” (*Re*) of the classification procedure to compute the score expressed by:

$$F-measure=2\times \frac{\mathrm{Pr}\times \text{Re}}{\mathrm{Pr}+\text{Re}}$$ (10)

The MI between estimated and true label is calculated by:

$$MI\left(\hat{C},C\right)=\sum_{\hat{c}=0}^{1}\sum_{c=0}^{1}p\left(\hat{c},c\right)\log\frac{p(\hat{c},c)}{p\left(\hat{c}\right)p\left(c\right)}$$ (11)

where $p(\hat{c},c)$ is the joint probability distribution function of estimated and true class labels $\hat{C}$ and *C*, and $p(\hat{c})$ and *p*(*c*) are the marginal probability distribution functions of $\hat{C}$ and *C* respectively.

The five-fold cross validation method is employed for experiments. Data samples are divided at random into five distinct subsets and four subsets are used for training classifiers whilst the last subset is for testing. For unbiased comparisons, each classifier is repeated 30 times and the average performance is reported along with the standard error.

To draw convincing conclusions in evaluating performance of feature selection methods and classifiers, we implement the Mann-Whitney U-test [54] for comparing two sets of results. The Mann-Whitney U-test is a nonparametric test of the null hypothesis that two populations have distributions with equal medians, against the alternative hypothesis that they do not. As the results over 30 trials may not be normally distributed, the use of Mann-Whitney U-test is more appropriate than that of normal-based methods [55].

Note that the test is performed to compare between the set of 30 outcomes generated by GRA method and that obtained by each of the competing feature selection methods using the same classifier. Similar procedure is performed to compare the GP classifier with its competing methods, i.e. kNN, MLP, SVM and AdaBoost using the same feature selection method.

## Results and Discussions

**Fig 5:**
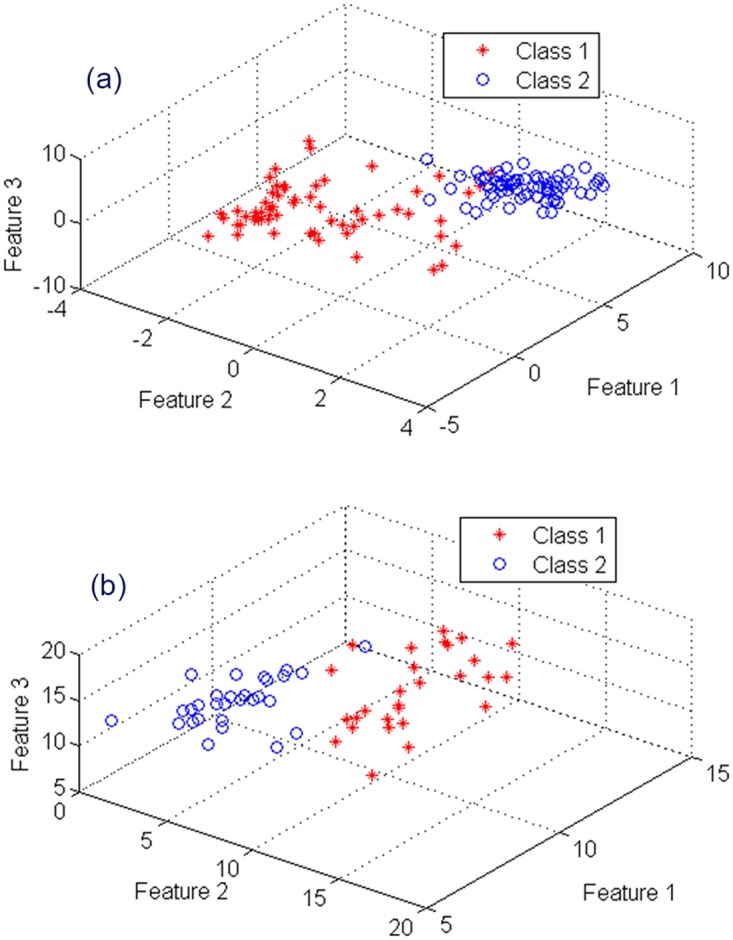
Distribution of data samples of the (a) Mont-Pick dataset and (b) cervical cancer dataset.

After voom transformation, GRA-based gene selection is employed for RNA-seq data to select genes that are differentially expressed for classification. GRA-based method performs as a filter method that ranks genes by combining outcomes of individual methods t-test, entropy, Bhattacharyya distance, Wilcoxon, and ROC. It therefore obtains the quintessence of these individual methods and provides stable and most discriminant subsets of genes. Fig 5 shows 3D presentations of feature subsets obtained by GRA-based method using the Mont-Pick and cervical cancer datasets. Obviously, GRA method is able to provide a clear separation between samples of two classes in both datasets. This facilitates the great classification performance of classifiers that use GRA-based feature subsets.

### Comparisons of GRA-based method with ReliefF, Simba, SNR, and IG

Feature subsets of top ten genes selected by GRA-based method serve as inputs into classifiers for demonstration although different number of genes can be used. For comparison, the same number of genes is selected by other methods in order to form feature subsets. Tables 2 and 3 present classification results of different feature selection methods for the Mont-Pick and cervical cancer datasets respectively. The classification is performed by the GP method and results for the accuracy, F-measure, AUC and MI metrics are reported in percentage.

**Table 2:** Results of feature selection methods using the Mont-Pick dataset (batch effect is addressed due to potentially different facilities).

Metrics	ReliefF	Simba	SNR	IG	GRA
Accuracy	93.78±0.80 (0.003)	84.81±1.26 (0.000)	96.05±0.49 (*0.154*)	90.80±1.22 (0.000)	96.77±0.71
F-measure	95.44±0.82 (0.022)	86.24±1.27 (0.000)	94.87±0.66 (0.003)	91.32±1.54 (0.001)	97.64±0.48
AUC	95.16±0.69 (0.005)	86.76±1.13 (0.000)	95.39±0.65 (0.031)	90.92±1.21 (0.000)	97.43±0.51
MI	75.89±3.15 (0.012)	42.99±3.60 (0.000)	78.88±2.46 (0.018)	72.68±4.73 (0.008)	88.56±2.71

**Table 3:** Results of feature selection methods using the cervical cancer dataset.

Metrics	ReliefF	Simba	SNR	IG	GRA
Accuracy	88.33±1.54 (0.013)	87.35±2.02 (0.029)	88.23±1.65 (0.031)	90.05±1.80 (*0.199*)	93.43±1.28
F-measure	87.92±1.67 (0.023)	84.95±2.74 (0.042)	87.67±1.70 (0.020)	90.77±1.58 (*0.280*)	92.91±1.57
AUC	88.23±1.47 (0.006)	87.61±1.98 (0.022)	89.21±1.58 (0.043)	91.89±1.38 (*0.316*)	94.07±1.22
MI	58.48±4.60 (0.024)	57.07±6.08 (0.044)	57.83±4.81 (*0.085*)	66.16±5.10 (*0.361*)	73.96±4.85

Each cell in these tables represents the mean and standard error of 30 classification outcomes. The value in brackets shows the *p*-value of the statistical Mann-Whitney U-test between each of the competing methods and the GRA method. For example, the value of 0.003 in the cell Accuracy-ReliefF in Table 2 is the *p*-value of the Mann-Whitney U-test between two sets of accuracy outcomes: one set is generated by using ReliefF and the other is obtained by GRA. The *p*-value smaller than 0.05 (the 5% significance level) indicates that the difference between two sets are statistically significant. In other words, the GRA method is significantly better than the ReliefF method. Values in italic in Tables 2 and 3 are *p*-values that are greater than 0.05.

In the Mont-Pick dataset, GRA shows a great performance compared with its competing methods. Specifically, GRA’s accuracy is of 96.77%, which is higher than ReliefF, Simba, SNR and IG of 93.78%, 84.81%, 96.05% and 90.80% respectively. Similar finding is seen in the cervical cancer dataset where GRA method outperforms other feature selection methods with regard to the accuracy metric. GRA obtains 93.43% of accuracy whilst those of ReliefF, Simba, SNR and IG are 88.33%, 87.35%, 88.23% and 90.05% respectively. Via other performance metrics, i.e. F-measure, AUC and MI, GRA feature selection method also demonstrates a considerable superiority to its competing methods. For example, in the Mont-Pick dataset, MI of GRA is of 88.56%, which is much greater than that of ReliefF at 75.89%, Simba at 42.99%, SNR at 78.88% and IG at 72.68%. Likewise, in the cervical cancer dataset, GRA’s MI is of 73.96%, which is the highest performance among the examined feature selection methods.

The GRA method obtains not only greater performance but also more stable results compared with its competing methods. This is demonstrated via standard errors of the results. In the Mont-Pick dataset, GRA results’ standard errors are mostly smaller than those of ReliefF, Simba, SNR and IG. In the cervical cancer dataset, GRA also obtains smaller standard errors than those of its competing methods except only one case MI-ReliefF.

Results of the Mann-Whitney U-test for evaluating feature selection methods demonstrate the statistical significance of GRA against its competing methods. In the Mont-Pick dataset, *p*-values of the Mann-Whitney U-test are smaller than 0.05 except only one case Accuracy-SNR. Therefore, the Mann-Whitney U-test rejects the null hypothesis that results of two methods (GRA and each of the competing feature selection methods) come from the same distribution at the 5% significance level. This means that GRA is significantly better than its competing methods in terms of all performance metrics. In the cervical cancer dataset, most *p*-values of the Mann-Whitney U-test are smaller than 0.05, except the comparisons of GRA with the IG method (for all performance metrics) and the SNR method (for MI metric).

### Comparisons of GP classifier with kNN, MLP, SVM, and AdaBoost

We use the GRA-based feature selection method to obtain subsets of top ten features that are fed into every classifier for comparisons. Results of all classifiers are presented in Tables 4 and 5 for the Mont-Pick and cervical cancer datasets respectively.

**Table 4:** Comparisons of classifiers using the Mont-Pick dataset (batch effect is addressed due to potentially different facilities).

Metrics	kNN	MLP	SVM	AdaBoost	GP
Accuracy	95.16±0.57 (0.019)	95.15±0.70 (0.038)	95.01±0.89 (*0.133*)	94.50±0.72 (0.026)	96.77±0.71
F-measure	96.09±0.52 (0.019)	94.19±0.87 (0.001)	93.97±0.94 (0.002)	94.99±0.66 (0.009)	97.64±0.48
AUC	96.78±0.52 (*0.473*)	94.93±0.55 (0.000)	94.50±0.96 (0.012)	95.30±0.64 (0.014)	97.43±0.51
MI	76.46±2.56 (0.007)	80.06±2.53 (0.018)	81.60±3.07 (*0.212*)	76.62±3.24 (0.028)	88.56±2.71

**Table 5:** Comparisons of classifiers using the cervical cancer dataset.

Metrics	kNN	MLP	SVM	AdaBoost	GP
Accuracy	88.11±1.53 (0.012)	85.33±2.00 (0.002)	87.42±2.21 (0.045)	88.38±1.32 (0.017)	93.43±1.28
F-measure	87.63±1.67 (0.016)	86.18±1.93 (0.011)	83.29±3.40 (0.019)	87.60±1.34 (0.005)	92.91±1.57
AUC	88.67±1.40 (0.007)	87.22±1.77 (0.006)	88.84±2.19 (*0.065*)	88.90±1.34 (0.010)	94.07±1.22
MI	57.49±4.53 (0.028)	51.53±5.24 (0.003)	60.29±5.43 (*0.126*)	57.47±4.08 (0.030)	73.96±4.85

Clearly, the GP classifier achieves greater performance compared with its competing methods in both datasets. The difference between GP with kNN, MLP, SVM and AdaBoost in the cervical cancer dataset is more considerable than that in the Mont-Pick dataset. The gap between GP and its competing methods is more than 5% in the cervical cancer dataset. More considerably, GP’s MI is greater than those of kNN, MLP, SVM and AdaBoost by more than 13%. MI of kNN, MLP, SVM and AdaBoost are respectively of 57.49%, 51.53%, 60.29% and 57.47%, which are much lower than 73.96% of the GP classifier.

With regard to the Mann-Whitney U-test results, *p*-values are almost smaller than 0.05, except three cases, AUC-kNN, Accuracy-SVM and MI-SVM, in the Mont-Pick dataset and two cases, AUC-SVM and MI-SVM, in the cervical cancer dataset.

### Comparisons of the proposed approach with sPLDA classifiers

In this subsection, we compare our approach, GP classifier using GRA-based feature subsets, with sparse Poisson linear discriminant analysis (sPLDA) classifiers, which were proposed by Witten [6]. The classification rule assigns the test observation x* to the class for which the following expression is largest:

$$\log P\left(\hat{y^{*}=k}|x^{*}\right)=\log{\hat{f}}_{k}\left(x^{*}\right)+\log{\hat{\pi }}_{k}+c=\overset{p}{\underset{j=1}{\sum }}X_{j}^{*}\log{\hat{d}}_{kj}-{\hat{s}}^{*}\overset{p}{\underset{j=1}{\sum }}{\hat{g}}_{j}{\hat{d}}_{kj}+\log{\hat{\pi }}_{k}+c^{\prime }$$ (12)

where *c* and *c*′ are constants that are not dependent on the class label, whilst ${\hat{\pi }}_{k}$ is the estimate of the prior probability that an observation belongs to class *k*. We set ${\hat{\pi }}_{1}=...={\hat{\pi }}_{K}=1/K$, corresponding to the prior that all classes are equally likely. Alternatively, ${\hat{f}}_{k}$ is an estimate of the density of an observation in class *k*. A Poisson model for RNA sequencing data states that $X_{j}^{*}|y^{*}=k\sim \text{Poisson}(s^{*}g_{i}d_{kj})$ where s* = *s*_1_, …, *s*_*n*_ are the size factors for the training data, which can be estimated using the total count, median ratio and quantile as follows:

Total count: ${\hat{s}}^{*}=\sum_{j=1}^{p}X_{j}^{*}/X..$ where *X*.. is the total number of counts of the training data.

Median ratio: ${\hat{s}}^{*}=m^{*}/\sum_{i=1}^{n}m_{i}$ where $m^{*}\;=\;{\text{median}}_{j}\left\{\frac{X_{j}^{*}}{{\left(\Pi_{i=1}^{n}\;X_{ij}\right)}^{1/n}}\right\}$ and $m_{i}\;=\;{\text{median}}_{j}\left\{\frac{X_{i}{}_{j}}{{\left(\Pi_{i^{\prime }=1}^{n}\;X_{i^{\prime }j}\right)}^{1/n}}\right\}$.

Quantile: ${\hat{s}}^{*}=q^{*}/\sum_{i=1}^{n}q_{i}$ where q* is the 75th percentile of counts for the test observation, and *q*_*i*_ is the 75th percentile of counts for the *i*th training observation.

**Fig 6:**
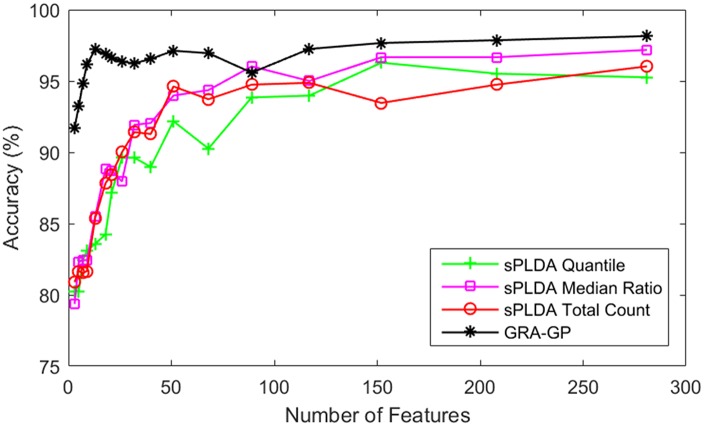
Comparisons of GRA-GP method with sPLDA classifiers using the Mont-Pick dataset.

**Fig 7:**
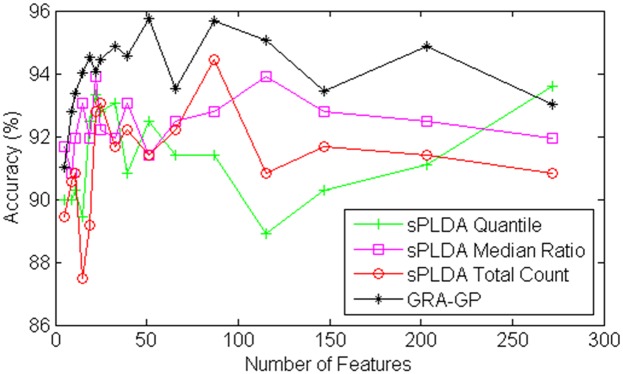
Comparisons of GRA-GP method with sPLDA classifiers using the cervical cancer dataset.

The estimate of *g*_*i*_ is given by ${\hat{g}}_{j}=X._{j}$ where $X._{j}=\sum_{i=1}^{n}X_{ij}$ and estimate of *d*_*kj*_ for sparse features is provided by:

$${\hat{d}}_{kj}=\left\{\begin{array}{c} \frac{a}{b}-\frac{\rho }{\sqrt{b}},\\ \frac{a}{b}+\frac{\rho }{\sqrt{b}},\\ 1, \end{array}\begin{array}{c} \text{if}\;\sqrt{b}\left(\frac{a}{b}-1\right)>\rho ,\\ \text{if}\;\sqrt{b}\left(1-\frac{a}{b}\right)>\rho ,\\ \text{if}\;\sqrt{b}\left|1-\frac{a}{b}\right|<\rho , \end{array}\right.$$ (13)

where $a=X_{C_{kj}}+\beta$, $b=\sum_{i\in C_{k}}{\hat{s}}_{i}{\hat{g}}_{j}+\beta$, and *ρ* is a nonnegative tuning parameter that is chosen by cross-validation. The number of features involved in classification is different when *ρ* is different. For unbiased comparisons, the number of features selected by our approach GRA-GP is the same with those determined by sPLDA. There are three approaches of sPLDA based on three corresponding methods of estimating the size factors for the training data, i.e. total count, median ratio and quantile. The comparisons are graphically depicted in Figs 6 and 7 by using the Mont-Pick and cervical cancer datasets respectively.

Results presented in these figures are obtained using the five-fold cross validation for each of the competing methods. We limit the number of features to approximately 300 and the performance is measured by accuracy in percentage. For each of the specified number of features, each classifier is repeated 30 times and the average result is reported. It is clear that GRA-GP method significantly dominates all three methods of sPLDA in both datasets based on different number of features. In the Mont-Pick dataset, the gaps between GRA-GP method with its competing methods are very large when the number of features are smaller than 100. GRA-GP is still constantly superior to sPLDA classifiers when the number of feature increases. In the cervical cancer dataset, there are small gaps between GRA-GP and its competing methods when the number of features are smaller than 25. These gaps are larger when the number of features increases. sPLDA median ratio relatively ranks as the second best method after the GRA-GP. This highlights the effectiveness of our approach against the sPLDA classifiers.

## Conclusions and Future Work

This paper proposes a new approach to RNA-seq count data classification using GRA-based feature selection method and the nonparametric GP models. RNA-seq data are assembled in integer read counts that present extreme values, high skewness, and heteroscedasticity. The voom transformation applied to RNA-seq data has turned them into microarray-like data by which a range of normal-based statistical methods can be utilized. On one hand, GRA systematically combines outcomes of individual methods, i.e. two-sample t-test, entropy test, Bhattacharyya distance, Wilcoxon test and ROC, and provides stable and robust feature subsets. By incorporating advantages and quintessence of the individual methods, GRA has shown a clear superiority to its competing methods that include ReliefF, Simba, signal to noise ratio, and information gain.

On the other hand, the nonparametric GP models based on the Bayesian inference methodology have addressed effectively the complexity of RNA-seq data. GP has demonstrated a considerable dominance in RNA-seq data classification against its competing methods including kNN, MLP, SVM and ensemble learning AdaBoost. Through analytic formulae, GP models are computationally tractable and easier to handle and interpret than their conventional counterparts such as neural networks. Via the characterization of mean and covariance functions, GP model fitting requires only the first- and second-order moments of the process to be specified. GP therefore has the generalization capability that has increased the classification performance. More considerably, the proposed GRA-GP approach has produced greater classification performance on different numbers of features against the sPLDA classifiers, which were proposed particularly for read counts modelling.

The use of benchmark real datasets along with the employment of various evaluation metrics, i.e. accuracy rate, F-measure, AUC and MI, ensure the findings of this research are well-justified. Application of the Mann-Whitney U-test has confirmed the statistical significance of the comparisons. This implies that the proposed approach can be implemented for many applications including finding potential markers of diseases, virus and bacteria type classification, and cancer prediction. Further work would be devoted to exploring different feature selection methods that may provide great performance specifically for count data classification. With the effectiveness in classifying RNA-seq data, GP models have demonstrated as a promising Bayesian approach in analysis of genomic data. Investigating Bayesian GP models to deal with challenges of other types of biological data is worth another future study.

## Supporting Information

S1 File voom transformation method. This file contains the description of the voom transformation method.
(PDF)

S2 File Details of the Gaussian process method. This file contains the details of the Gaussian process method.
(PDF)

S3 File Graphical comparisons of feature selection methods and classifiers. This file contains the graphical comparisons by box plots of feature selection methods and classifiers.
(PDF)
