# Supplementary material for: RNA-Seq Count Data Modelling by Grey Relational Analysis and Nonparametric Gaussian Process
Source: PLoS One. 2016 Oct 26;11(10):e0164766. doi: 10.1371/journal.pone.0164766 (PMC5082617; doi:10.1371/journal.pone.0164766)
Supplement: S1 File — This file contains the description of the voom transformation method. (PDF) [file pone.0164766.s001.pdf]

voom transformation method

Assume that RNA-seq data is organized as a matrix of read counts r_{gi} where sample index $i = 1$ to n and genes $g = 1$ to G. The voom algorithm first defines the log-counts per million (log-cpm) value for each count as:

$$y_{gi} = \log_2 \left(\frac{r_{gi} + 0.5}{R_i + 1} \times 10^6 \right) \tag{1}$$

where R_i denotes the total number of mapped reads for sample i, $R_i = \sum_{g=1}^{G} r_{gi}$.

In the second step, voom tries to fit RNA-seq data by a linear model:

$$E(y_{gi}) = \mu_{gi} = x_i^T \beta_g \tag{2}$$

where x_i is a vector of covariates and β_g is a vector of unknown coefficients representing $\log_2$-fold-changes. The above model is applied to the log-cpm values y_{gi} for each gene. After modelling, regression coefficient estimates $\hat{\beta}^*_{gi}$ can be obtained along with the fitted values $\hat{\mu}_{gi} = x_i^T \hat{\beta}_g$.

Next the fitted log-cpm valules $\hat{\mu}_{gi}$ are converted to fitted counts by:

$$\hat{\lambda}_{gi} = \hat{\mu}_{gi} + \log_2(R_i + 1) - \log_2(10^6) \tag{3}$$

The voom precision weights are then calculated as the inverse variances w_{gi} =lo$(\hat{\lambda}_{gi})^{-4}$ where lo is a piecewise linear function defined by the locally weighted regression curve [1].

References

[1] Law CW, Chen Y, Shi W, Smyth GK. Voom: precision weights unlock linear model analysis tools for RNA-seq read counts. Genome Biology. 2014 Feb 3;15(2):1.
