# Supplementary material for: RNA-Seq Count Data Modelling by Grey Relational Analysis and Nonparametric Gaussian Process
Source: PLoS One. 2016 Oct 26;11(10):e0164766. doi: 10.1371/journal.pone.0164766 (PMC5082617; doi:10.1371/journal.pone.0164766)
Supplement: S2 File — This file contains the details of the Gaussian process method. (PDF) [file pone.0164766.s002.pdf]

Details of the Gaussian process method

Given a model specification (i.e., GP mean and covariance functions and a likelihood function) and a data set $D = \{(y_i, x_i)|i = 1, 2, ..., m\}$, the inference method can compute the approximate posterior, the approximate negative log marginal likelihood and its partial derivatives with regard to the hyperparameters. Kuss and Rasmussen [1] recommended the EP approximate method over the Laplace's method for binary classification problems. Moreover, the MCMC sampling method is computationally expensive. Accordingly, the EP method is chosen for experiments in this study.

The principle of EP method is to approximate the non-Gaussian likelihood terms $p(y_i|f_i)$ by Gaussian functions $t(f_i; \nu_i, \tau_i) = \exp(\nu_i f_i - \frac{1}{2}\tau_i f_i^2)$ so that the following condition is satisfied:

$$\frac{1}{Z_{t,i}} \int f^k q_{-i}(f).t(f_i; \nu_i, \tau_i) df = \frac{1}{Z_{p,i}} \int f^k q_{-i}(f).p(y_i|f) df, \qquad k = 1, 2 \tag{1}$$

where $Z_{t,i}$ and $Z_{p,i}$ are two normalizers specified by:

$$Z_{t,i} = \int q_{-i}(f).t(f_i; \nu_i, \tau_i) df \quad \text{and} \quad Z_{p,i} = \int q_{-i}(f).p(y_i|f_i) df \tag{2}$$

and $q_{-i}(f)$ is the cavity distribution is defined as:

$$q_{-i}(f) = \mathbb{N}(f|m, K) \prod_{j \neq i} t(f_i; \nu_i, \tau_i) \propto \mathbb{N}(f|\mu, V)/t(f_i; \nu_i, \tau_i). \tag{3}$$

Hyperparameters of a GP comprise three components: mean, covariance and likelihood function. A mean function m_Φ with hyperparameters Φ of a GP f specified over the whole domain X as $m_\Phi : X \to R$ is a scalar function that computes the expected value $m(x) = E[f(x)]$ of f for the input x. We choose the linear mean function because the RNA-seq count data after voom transformation can be processed by linear models [2]. The linear function is defined as $m(x) = a^T x$, with $x \in X \subseteq R^D$ and $a \in R^D$. In this paper, a is set as a vector of values that are all equal to 1.

A covariance function $k_\psi : X \times X \to R$ of a GP f is a scalar function specified over the whole domain X^2 that calculates the covariance $k(x, x\prime) = V[f(x), f(x')] = E[(f(x) - m(x))(f(x') - m(x'))]$ between inputs x and x'. The squared exponential covariance function with automatic relevance determination (ARD) is investigated for experiments:

$$k(x, x', \theta) = \sigma^2 \exp\left(-\frac{1}{2}(x - x')^T M (x - x')\right) \tag{4}$$

where σ^2 is the signal variance and $M = diag(l)^{-2}$ is a diagonal matrix that comprises ARD parameters. The l parameter plays the role of characteristic length-scales, which constitute a vector of positive values. In the experiments, we set $\sigma = 1$ and l is a vector of values that are all equal to 1.

A likelihood function $p(y|f)$ is a conditional density $\int p(y|f) dy = 1$ relating the scalar latent function values f and outputs y. In this study, the error

likelihood function is chosen in the form $p(y_i|f_i) = \int_{-\infty}^{y_i f_i} \mathbb{N}(t)dt$. Given the latent function, the joint likelihood is given by:

$$p(y|f) = \prod_{i=1}^{m} p(y_i|f_i) \tag{5}$$

because the class labels are independent Bernoulli variables. There are several ways to transform function values to class probabilities using squashing functions: sigmoid (logistic), cumulative normal (probit), threshold or robust threshold. We use the probit model $p(y = 1|x) = \Phi(f(x))$ where Φ specifies the cumulative density function of the standard Gaussian distribution. As Φ is symmetric, the individual likelihood terms is expressed by $p(y_i|f_i) = \Phi(y_i f_i)$.

GP generates a prediction at x_* conditioned on the data $D = (X, y)$ by generating the predictive mean μ_{y_*} and variance $\sigma^2_{y_*}$. These values are calculated from the latent Gaussian marginal approximation $\mathbb{N}(f_*|\mu_{f_*}, \sigma^2_{f_*})$ through two moments μ_{f_*} and $\sigma^2_{f_*}$. Using GP, the test predictions are in the form of class probabilities, which contrasts with approaches producing only a guess at the class label. The approximation yields:

$$p(y_*|D, x_*) = \int p(y_*|f_*)p(f_*|D, x_*)df_* \approx \int p(y_*|f_*)\mathbb{N}(f_*|\mu_{f_*}, \sigma^2_{f_*})df_* \tag{6}$$

The predictive moments are then computed by $\mu_{y_*} = \int y_* p(y_*|D, x_*)dy_*$ and $\sigma^2_{y_*} = \int (y_* - \mu_{y_*})^2 p(y_*|D, x_*)dy_*$.

A binary classification problem is characterized by $y_* \in \{-1, +1\}$ and $p_{y_*} + p_{-y_*} = 1$. Using $\pi_* = p_{+1}$, we find that $p_{y_*} = \pi_*$ if $y_* = +1$ and $p_{y_*} = 1 - \pi_*$ when $y_* = -1$. Therefore, μ_{y_*} and $\sigma^2_{y_*}$ can be calculated by:

$$\mu_{y_*} = \sum_{y_* = \pm 1*} y_* p(y_*|D, x_*) = 2\pi_* - 1 \in [-1, 1] \tag{7}$$

$$\sigma^2_{y_*} = \sum_{y_* = \pm 1*} (y_* - \mu_{y_*})^2 p(y_*|D, x_*) = 4\pi_*(1 - \pi_*) \in [0, 1] \tag{8}$$

References

[1] Kuss M, Rasmussen CE. Assessing approximate inference for binary Gaussian process classification. Journal of Machine Learning Research. 2005;6(Oct):1679-704.

[2] Law CW, Chen Y, Shi W, Smyth GK. Voom: precision weights unlock linear model analysis tools for RNA-seq read counts. Genome Biology. 2014 Feb 3;15(2):1.
