# Supplementary material for: RNA-Seq Count Data Modelling by Grey Relational Analysis and Nonparametric Gaussian Process
Source: PLoS One. 2016 Oct 26;11(10):e0164766. doi: 10.1371/journal.pone.0164766 (PMC5082617; doi:10.1371/journal.pone.0164766)
Supplement: S3 File — This file contains the graphical comparisons by box plots of feature selection methods and classifiers. (PDF) [file pone.0164766.s003.pdf]

Graphical comparisons of feature selection methods and classifiers

Figs A and B show box plots for graphical comparisons of feature selection methods using the Mont-Pick and cervical cancer datasets respectively. In each dataset, there are four plots representing results in terms of accuracy, F-measure, AUC and MI. Each box in these plots presents the median and distribution of 30 outcomes. In line with the results reported in Tables 2 and 3 (in the main text), GRA-based feature selection method dominates ReliefF, Simba, SNR and IG in all performance metrics. This is because the median values of the GRA boxes are greater than those of other methods in both datasets.

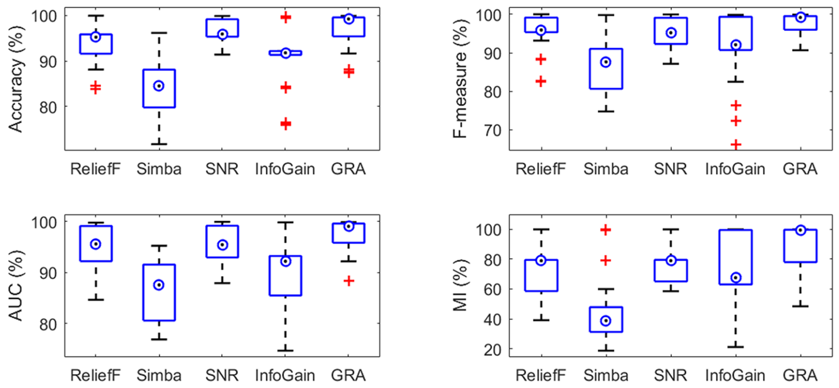

Fig A. Comparisons of feature selection methods in terms of accuracy, F-measure, AUC, and MI by box plots using the Mont-Pick dataset.

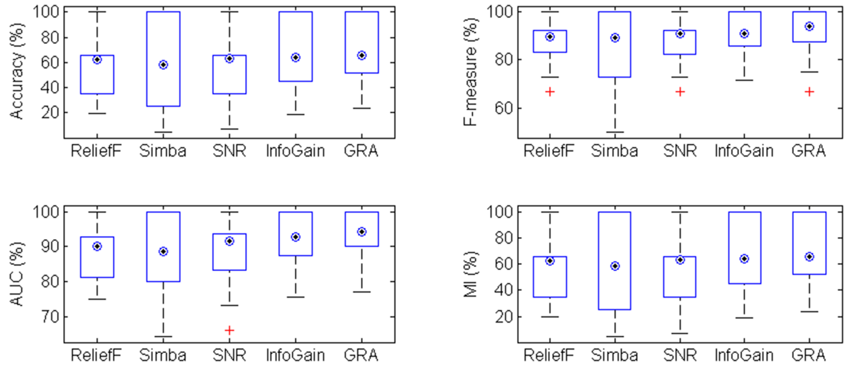

Fig B. Comparisons of feature selection methods in terms of accuracy, F-measure, AUC, and MI by box plots using the cervical cancer dataset.

Box plots for comparisons of classifiers using the Mont-Pick and cervical cancer datasets are shown in Figs C and D respectively. The median values of

the GP boxes are greater than those of kNN, MLP, SVM and AdaBoost. This is consistent with the average values presented in Tables 4 and 5 (in the main text). Moreover, GP also generates relatively stable results compared with other methods. The interquartile ranges of GP boxes are among smallest ones of the five classification methods. In the cervical cancer dataset, boxes of GP, kNN and AdaBoost are smaller than those of MLP and SVM in terms of accuracy, F-measure and AUC. With regard to the MI metric, GP box is larger than those of kNN, MLP and AdaBoost but it has the smallest minimum-maximum range among five feature selection methods.

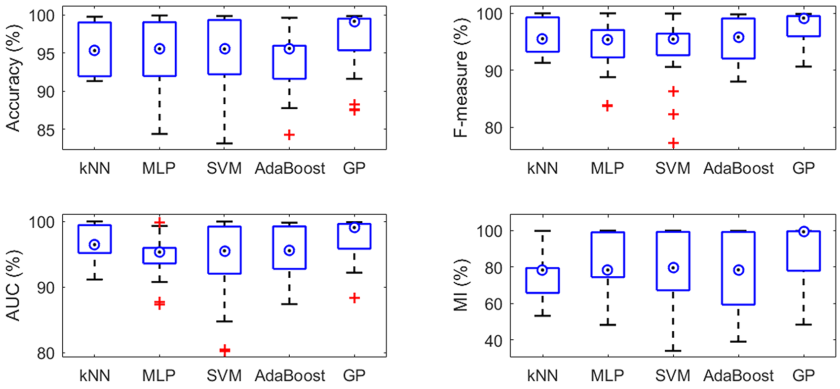

Fig C. Comparisons of classifiers in terms of accuracy, F-measure, AUC and MI by box plots using the Mont-Pick dataset.

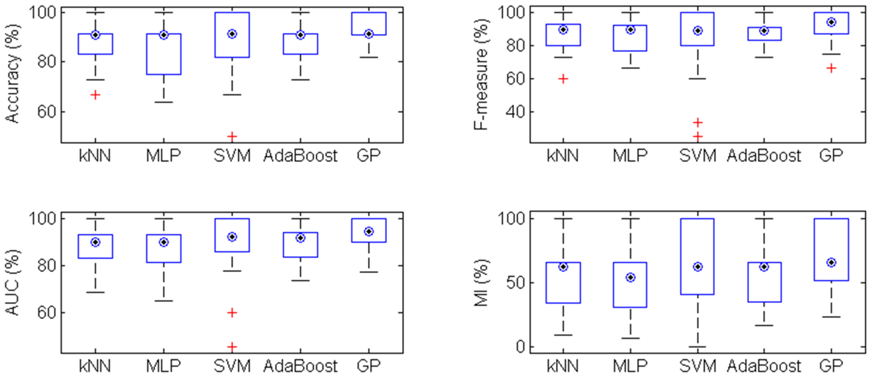

Fig D. Box plots for comparisons of classifiers in terms of accuracy, F-measure, AUC and MI using the cervical cancer dataset.
